# Supplementary material for: Identification of photoperiod-induced specific miRNAs in the adrenal glands of Sunite sheep (Ovis aries)
Source: Front Vet Sci. 2022 Jul 22;9:888207. doi: 10.3389/fvets.2022.888207 (PMC9354845; doi:10.3389/fvets.2022.888207)
Supplement: Supplementary file 1 [file Data_Sheet_1.ZIP › Supplementary file/miRDeep2 file.zip!/miRDeep2 file/Novel_Structure/SPLP42A1/Novel_668.pdf]

Provisional ID	:	NC_019481.2_30078
Score total	:	2995.2
Score for star read(s)	:	3.9
Score for read counts	:	2987.7
Score for mfe	:	2
Score for randfold	:	1.6
Score for cons. seed	:	
Total read count	:	5872
Mature read count	:	5855
Loop read count	:	0
Star read count	:	17

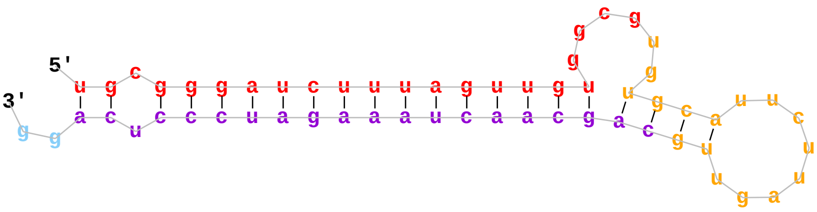

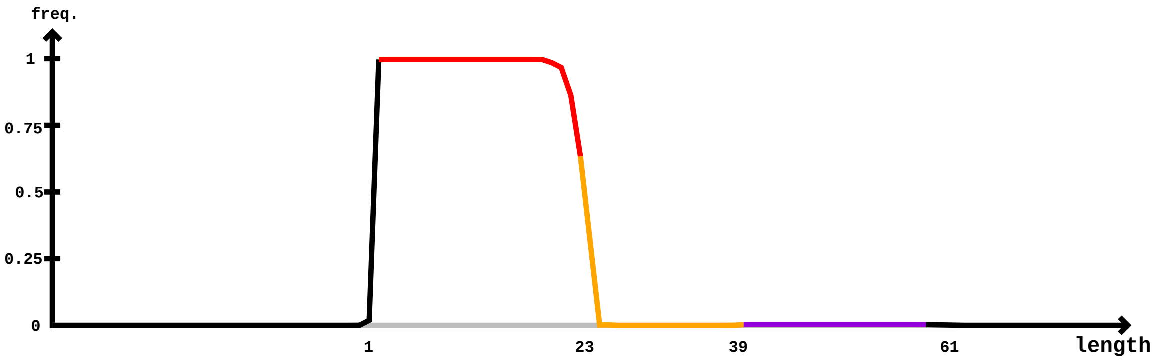


```
                                         Mature                                 Star
5'- aauaacccauaaaaagcagccgacguugcggcaugcgggaucuuuaguuguggcgugugcauucuuaguugcagcaacuaaagaucccucaggcugcaaugaagaucagag   -3'    obs
    aauaacccauaaaaagcagccgacguugcggcaugcgggaucuuuaguuguggcgugugcauucuuaguugcagcaacuaaagaucccucaggcugcaaugaagaucagag          exp
    ....................(..(((((((((.((.(((((((((((((((......((((........))))))))))))))))))).)).)))))))))..).......    reads   mm   sample
    ...............................caugcgggaucuuuaguugugg..........................................................    1       0    seq
    ...............................caugcgggaucuuuaguuguggcA........................................................    1       1    seq
    ................................Cugcgggaucuuuaguugu............................................................    1       1    seq
    ................................augcgggaucuuuaguugG............................................................    2       1    seq
    ................................augcgggaucuuuaguugA............................................................    1       1    seq
    ................................augcgggaucuuuaguugC............................................................    2       1    seq
    ................................augcgggaucuuuaguugugg..........................................................    13      0    seq
    ................................augcgggaucuuuaguugugU..........................................................    1       1    seq
    ................................augcgggaucuuuaguuguggc.........................................................    44      0    seq
    ................................auUcgggaucuuuaguuguggc.........................................................    2       1    seq
    ................................augcgggaucuuuaguuguggU.........................................................    19      1    seq
    ................................auAcgggaucuuuaguuguggc.........................................................    1       1    seq
    ................................augcgggaucuuuaguuguggcC........................................................    1       1    seq
    ................................augcgggaucuuuaguuguggcA........................................................    21      1    seq
    .................................ugcgggaucuuuaguug.............................................................    2       0    seq
    .................................ugcgggaucuuuaguugA............................................................    7       1    seq
    .................................ugcgggaucuuuaguugu............................................................    56      0    seq
    .................................uAcgggaucuuuaguugug...........................................................    1       1    seq
    .................................ugcgggaucuuuaguuguA...........................................................    28      1    seq
    .................................ugcAggaucuuuaguugug...........................................................    2       1    seq
    .................................ugcgggaucuuuaguugug...........................................................    76      0    seq
    .................................uAcgggaucuuuaguugugg..........................................................    3       1    seq
    .................................ugcgggaucuuuaguugugA..........................................................    137     1    seq
    .................................ugcAggaucuuuaguugugg..........................................................    8       1    seq
    .................................ugcgggaucuuuaguugCgg..........................................................    1       1    seq
    .................................ugcgggaucuuuaguugugg..........................................................    422     0    seq
    .................................ugcgggaucuuuaguugugU..........................................................    24      1    seq
    .................................ugcAggaucuuuaguuguggc.........................................................    14      1    seq
    .................................ugcgggaucuuuaguuguggc.........................................................    869     0    seq
    .................................uAcgggaucuuuaguuguggc.........................................................    6       1    seq
    .................................ugcgggaucuuuaguuguggU.........................................................    390     1    seq
    .................................uAcgggaucuuuaguuguggcg........................................................    19      1    seq
    .................................ugcAggaucuuuaguuguggcg........................................................    26      1    seq
```

```
                                Mature                                Star
aauaacccauaaaagcagccgacguugcggcaugcgggaucuuuaguuguggcgugugcauucuuaguugcagcaacuaaagaucccucaggcugcaaugaagaucagag
................................ugcgggaucuuuaguuguggcC........................................................  62    1  seq
................................ugcgggaucuuuagCuguggcg........................................................  9     1  seq
................................ugcgggGucuuuaguuguggcg........................................................  13    1  seq
................................ugcgggaucuuuaguuguggcg........................................................  3530  0  seq
................................ugcgggaucuuuagCuguggcgu.......................................................  4     1  seq
................................ugcgggaucuuuaguuguggcCu.......................................................  10    1  seq
................................uAcgggaucuuuaguuguggcgu.......................................................  7     1  seq
................................ugcgggGucuuuaguuguggcgu.......................................................  9     1  seq
................................ugcAggaucuuuaguuguggcgug......................................................  1     1  seq
................................ugcgggaucuuuaguuguggcgugu.....................................................  8     0  seq
.................................gcgggaucuuuaguuguggc.........................................................  1     0  seq
....................................................................ugcagcaacuaaagaucccA......................  1     1  seq
......................................................................cagcaacuaaagaucccuca....................  10    0  seq
......................................................................cagcaacuaaagaucccucaggc.................  4     0  seq
.......................................................................agcaacuaaagaucccucaggc.................  2     0  seq
```
